# Hybrid near-infrared and chemical-based machine learning enhances the reliability of chili powder origin classification

**DOI:** 10.1038/s41598-026-47486-7

**Published:** 2026-04-06

**Authors:** Ji-Hee Yang, Hae-Il Yang, Se-Jin Park, Sung-Gi Min, Woo Jin Jun, Young-Bae Chung

**Affiliations:** 1 Kimchi Factory Research Group, World Institute of Kimchi, Gwangju, 61755 Republic of Korea; 2 Division of Food and Nutrition, and Research Institute for Human Ecology, Chonnam National University, Gwangju, 61186 Republic of Korea

**Keywords:** Origin classification, Spectral preprocessing, Feature selection, Probability reliability, SHAP analysis, Machine learning interpretability, Chemistry, Materials science

## Abstract

Accurate origin classification of chili powder is essential for consumer trust and regulatory compliance. In this study, combined near-infrared (NIR) spectroscopy and chemical composition analysis were integrated with machine learning to classify domestic (n = 54, Korea) and imported (n = 66, China and Vietnam) chili powder samples. Baseline analysis revealed systematic differences: domestic powders showed higher protein, calcium, and moisture contents, whereas imported samples contained more organic acids, sugars, and capsaicinoids. Using 16 NIR bands selected by the least absolute shrinkage and selection operator (LASSO), support vector machine (SVM) models achieved high accuracy, with Savitzky–Golay first derivative plus standard normal variate preprocessing yielding the best performance. The hybrid models enhanced reliability. NIR alone achieved high origin-classification accuracy in this dataset using as few as four selected bands; however, NIR combined with organic acid variables (NIR + org) consistently achieved 100% accuracy and showed improved probability reliability. Shapley additive explanation analysis showed that while O–H and C–H overtone bands drove the NIR spectral band-only models, the hybrid models emphasized organic acids and proximate components, providing clear chemical interpretability. The findings demonstrated that integrating NIR with targeted chemical variables enables robust, reliable, and interpretable origin classification, offering rapid screening and regulatory assurance.

**Supplementary Information:**

The online version contains supplementary material available at 10.1038/s41598-026-47486-7.

## Introduction

Chili powder is one of the most widely consumed spices worldwide and a key ingredient in many traditional foods. Its sensory properties—particularly color, pungency, and flavor—are strongly influenced by its chemical composition, including capsaicinoids, organic acids, sugars, and pigments^1–3^. Consequently, the quality and safety of chili powder are closely linked to its compositional profile^4,5^, which can be affected by cultivar, growing environment, processing, and potential adulteration. Several studies have reported intentional adulteration of chili powder with non-permitted dyes (e.g., Sudan I–IV) and other fillers, raising serious regulatory and health concerns^6–8^.

Within this context, the geographical origin of chili powder is recognized as a key factor in origin labeling and in preventing misrepresentation in the market^9^. Origin is associated with systematic differences in capsaicinoids^10^, organic acids^11^, and mineral content^12^, which influence consumer perception and market value. In Korea, distinguishing between domestic and imported chili powder (mainly Chinese and Vietnamese) is especially important given the cultural and economic significance of kimchi production, in which chili powder is a primary component^9^. These issues underscore the need for rapid screening tools that can support origin-label compliance monitoring of chili powder quality and safety.

Various analytical approaches have been proposed to classify the origin of chili powder. Conventional chemical analyses, such as electronic nose profiling^13^, organic acid composition^14^, and mineral content^9^, have demonstrated potential to distinguish origins. Advanced instrumental techniques, including femtosecond laser ablation–inductively coupled plasma–mass spectrometry^15^, nuclear magnetic resonance spectroscopy^4,16^, and chromatographic determination of color pigments using high-performance liquid chromatography (HPLC)^17^, have also been applied. However, many of these techniques require specialized instrumentation or non-routine procedures, which can limit their practicality for large-scale screening. Alternatively, spectroscopic techniques, such as near-infrared (NIR) and mid-infrared spectroscopy, have been widely used as rapid and nondestructive tools for food analysis^18^. While mid-IR spectroscopy can be advantageous for more straightforward chemical interpretation because it is dominated by fundamental vibrations, the NIR wavelength range (700–2500 nm) offers deeper penetration into powdered samples, enabling more representative measurements. In addition, NIR requires minimal sample preparation and can be readily implemented for on-line or at-line measurements, providing a cost-effective front-line option for high-throughput screening of the large numbers of chili powder batches that must be checked for origin labeling^19^. Nonetheless, the robustness of NIR-based models may be limited by broad and overlapping absorption bands, scattering effects, and noise^20^, highlighting the need for strategies that strengthen reliability for industrial adoption.

In the context of NIR-based food analysis, traditional hard-modelling (discriminant) approaches such as PLS-DA have been widely used, and more recently supervised machine-learning models have been applied to improve the classification of spectroscopic and compositional datasets^21,22^. Machine-learning models, particularly when combined with feature selection strategies, can achieve high accuracy in origin classification. Nevertheless, two key limitations remain: First, most prior studies have emphasized accuracy metrics alone, without considering probability reliability (e.g., expected calibration error, Brier score, and log loss), which is essential for assessing the reliability of model predictions^23^. Second, the interpretability of ML-based models is often limited, hindering their adoption in regulatory and industrial contexts, where transparent decision-making is required^24,25^.

To address these gaps, we adopt an integrative framework that combines spectral and chemical domains. Chemical variables provide direct and relatively noise-resistant measurements of composition, whereas NIR spectra offer rapid but less robust proxies influenced by scattering and spectral overlap. By incorporating a small number of carefully selected chemical markers and jointly optimizing the most informative NIR wavelengths, we construct hybrid models that balance analytical speed with improved robustness and mechanistic interpretability. We further apply Shapley additive explanations (SHAP) to quantify the contribution of each spectral and chemical feature, thereby linking model outputs to meaningful compositional traits^25^. To our knowledge, such a reliability-focused hybrid framework has not been explored for chili powder origin classification, particularly in an industrially scalable screening context.

This study aimed to develop and evaluate a comprehensive framework for chili powder origin classification by integrating NIR spectroscopy with chemical composition data. Specifically, we (i) characterized the baseline compositional differences between domestic and imported chili powders; (ii) compared spectral preprocessing and feature selection strategies for NIR-based classification; (iii) assessed the performance, probability reliability, and stability of NIR-only, chemical-only, and hybrid models; and (iv) employed SHAP analysis to interpret the most influential features across models. Compared with previous studies—which have largely relied on either chemical markers or spectroscopic fingerprints and have emphasized accuracy alone—this work is distinguished by its hybrid NIR–chemical models, explicit evaluation of probability reliability (ECE, Brier score, log loss), and SHAP-based interpretation of both spectral and chemical contributions, providing a transparent and practically relevant framework for origin classification and screening of chili powder.

## Results and discussion

### Baseline differences in chemical composition

Substantial compositional differences were observed between domestic (n = 54) and imported (n = 66) chili powder samples (Table 1). For example, imported products exhibited significantly higher a* and b* values, indicating a more intense red–yellow color, whereas domestic products had higher L* values, reflecting a lighter appearance. Moisture content was markedly higher in domestic samples (8.71 ± 0.92 vs. 6.58 ± 2.57, *p* < 0.001) than in imported samples, whereas imported samples showed significantly greater contents of organic acids such as citric acid (33,487 ± 6,248 vs. 18,742 ± 18,168 mg/kg, *p* < 0.001) and malic acid (15,049 ± 6,509 vs. 7,811 ± 7,515 mg/kg, *p* < 0.001). Sugars, including glucose, fructose, and total sugar, were also elevated in the imported samples, whereas sucrose levels were higher in the domestic products. Capsaicinoid levels (capsaicin and dihydrocapsaicin) were consistently higher in the imported samples, accounting for their stronger pungency. In contrast, the domestic powders had significantly higher protein and calcium contents, whereas the imported samples contained higher amounts of sodium and iron. These systematic differences provide a chemical basis for geographical origin classification and support the relevance of the NIR and hybrid models, which leverage such underlying contrasts.

**Table 1:** Comparison of physicochemical properties, organic acids, sugars, capsaicinoids, and mineral contents between domestic and imported chili powder samples.

	Domestic (Korea, n = 54)	Imported (China & Vietnam, n = 66)	*p* value	Significance
L*	35.79 ± 2.01	37.44 ± 5.56	0.028	*
a*	27.73 ± 5.05	29.84 ± 5.63	0.033	*
b*	20.85 ± 8.88	28.01 ± 12.47	< 0.001	***
Moisture content (%)	8.71 ± 0.92	6.58 ± 2.57	< 0.001	***
ASTA color	101.67 ± 24.46	111.82 ± 44.15	0.114	ns
Protein (mg/kg)	15.67 ± 1.54	13.75 ± 1.68	< 0.001	***
Fat (mg/kg)	10.23 ± 1.22	11.43 ± 3.29	0.007	**
Citric acid (g/kg)	18.74 ± 18.17	33.49 ± 6.25	< 0.001	***
Malic acid (g/kg)	7.81 ± 7.52	15.05 ± 6.51	< 0.001	***
Fumaric acid (mg/kg)	128.04 ± 142.06	101.34 ± 79.26	0.221	ns
Sucrose (g/kg)	4.27 ± 1.61	2.57 ± 1.27	< 0.001	***
Glucose (g/kg)	54.86 ± 19.24	73.05 ± 26.58	< 0.001	***
Fructose (g/kg)	105.34 ± 28.73	123.74 ± 33.34	0.002	**
Total sugar (g/kg)	164.47 ± 47.76	199.36 ± 57.96	< 0.001	***
Sodium (mg/100 g)	11.16 ± 5.91	1,105.22 ± 1,095.17	< 0.001	***
Calcium (mg/100 g)	1,354.86 ± 1,284.95	43.01 ± 46.30	< 0.001	***
Iron (mg/100 g)	4.54 ± 0.87	36.75 ± 30.25	< 0.001	***
K (mg/100 g)	1,656.39 ± 1,592.64	1,355.82 ± 1,376.34	0.277	ns
Mg (mg/100 g)	196.25 ± 10.81	87.36 ± 83.65	< 0.001	***
Capsaicin (g/kg)	314.19 ± 414.47	800.28 ± 859.61	< 0.001	***
Dihydrocapsaicin (g/kg)	235.33 ± 252.25	540.82 ± 637.29	0.001	***
Total capsaicin (g/kg)	549.53 ± 633.93	1,341.09 ± 1,480.06	< 0.001	***

Values are expressed as mean ± standard deviation. *p* values were obtained using Student’s *t*-test. ns, not significant; *p < 0.05, **p < 0.01, and ***p < 0.001.

Because the imported set spans multiple countries (China and Vietnam), we additionally summarized key variables by country (Table S2) and explored country-level similarity using a PCA score plot based on standardized compositional variables (Fig. S1). The PCA score plot (PC1 + PC2 = 52.8% variance) indicated country-level structure, with Vietnamese samples forming a distinct cluster along PC1, whereas Chinese and Korean samples showed partial overlap. Given the smaller number of Vietnamese samples (n = 9), the observed tight clustering for Vietnam should be interpreted cautiously.

### Effects of preprocessing on NIR spectral structure

**Fig. 1:**
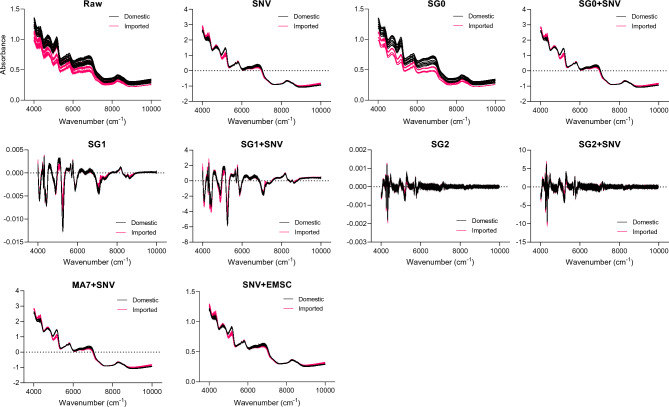
Raw and preprocessed NIR spectra of domestic (black) and imported (red) chili powder samples. Each panel represents a different preprocessing algorithm.

Figure 1 shows the raw and preprocessed NIR spectra for all domestic and imported samples, illustrating how different preprocessing algorithms modify the spectral structure. In the raw spectra, the two groups exhibited broadly similar profiles with substantial overlap, along with noticeable noise and baseline variation, although minor group-level differences appeared in specific spectral regions. Scatter-normalization algorithms such as SNV reduce multiplicative and additive scattering effects, minimizing variability caused by sample packing or particle-size differences. Derivative-based preprocessing (SG1 and SG2) enhances peak resolution by sharpening overlapping bands and removing slow-varying background components, thereby making subtle inter-group differences more visible. Combined algorithms (e.g., SG1 + SNV and SG2 + SNV) further stabilize the spectral profiles by simultaneously addressing baseline distortions, scattering variation, and overlapping features.

Several characteristic NIR absorption regions were consistently observed across samples. In the 4100–4800 cm^-1^ region, broad combination bands corresponding to O–H stretching and bending vibrations were identified, which are typically associated with water and other hydrogen-bonded groups in food matrices^26^. The 5800–6200 cm^-1^ region showed C–H first-overtone absorptions linked to sugars and other CH-rich constituents, whereas the 8300–8800 cm^-1^ region corresponded to second overtones of CH₂ and CH₃ stretching, as commonly reported in carbohydrate- and lipid-rich matrices^27^.

### Performance of spectral preprocessing algorithms

Using the 16 NIR bands selected by LASSO, we evaluated the performance of SVM models using different spectral preprocessing algorithms (Table 2). Among them, SG1 + SNV achieved the highest performance across all metrics (ACC 0.983 ± 0.037, F1 0.985 ± 0.034, Recall 1.000 ± 0.000, AUC 1.000 ± 0.000). Comparable but slightly lower performance was obtained with SNV + EMSC (ACC 0.962 ± 0.062), SG1 (0.971 ± 0.064), and SG2 (0.971 ± 0.064), whereas the raw spectra showed markedly inferior results (ACC 0.870 ± 0.132, Recall 0.833 ± 0.236). Notably, the AUC values remained consistently high across all preprocessing algorithms (0.983–1.000), indicating robust classification, whereas the differences were mainly observed in terms of accuracy and recall. The distribution of the optimal regularization parameter (C) further reflected the effect of preprocessing: SG1 + SNV required a relatively low value (0.4 ± 0.3), in contrast to the raw spectra and SG0 (3.0 ± 0.0). This suggests that SG1 + SNV effectively reduced spectral variability, enabling stronger regularization while maintaining stable classification margins. Overall, the SG1 + SNV consistently demonstrated superior predictive performance for distinguishing between domestic and imported samples, even under band-reduced conditions, and was therefore adopted as the standard preprocessing algorithm in subsequent analyses.

**Table 2:** Classification performance of support vector machine (SVM) models (16 NIR bands, least absolute shrinkage and selection operator (LASSO) feature selection) with different spectral preprocessing algorithms.

Method	ACC	F1	Precision	Recall	AUC	SVM_C_
Raw	0.870 ± 0.132	0.852 ± 0.159	0.910 ± 0.124	0.833 ± 0.236	0.983 ± 0.037	3.0 ± 0.0
SNV	0.946 ± 0.056	0.949 ± 0.051	0.940 ± 0.089	0.967 ± 0.075	0.996 ± 0.009	1.9 ± 1.5
SNV + EMSC	0.962 ± 0.062	0.961 ± 0.062	0.980 ± 0.045	0.950 ± 0.112	1.000 ± 0.000	1.0 ± 1.2
SG0	0.946 ± 0.056	0.948 ± 0.051	0.960 ± 0.089	0.944 ± 0.079	0.997 ± 0.006	3.0 ± 0.0
SG0 + SNV	0.954 ± 0.058	0.953 ± 0.058	0.965 ± 0.049	0.950 ± 0.112	1.000 ± 0.000	1.1 ± 1.1
MA7 + SNV	0.954 ± 0.058	0.953 ± 0.058	0.965 ± 0.049	0.950 ± 0.112	1.000 ± 0.000	1.1 ± 1.1
SG1	0.971 ± 0.064	0.978 ± 0.050	0.960 ± 0.089	1.000 ± 0.000	0.994 ± 0.012	2.2 ± 1.1
**SG1 + SNV**	**0.983 ± 0.037**	**0.985 ± 0.034**	0.971 ± 0.064	**1.000 ± 0.000**	**1.000 ± 0.000**	**0.4 ± 0.3**
SG2	0.971 ± 0.064	0.971 ± 0.064	1.000 ± 0.000	0.950 ± 0.112	0.996 ± 0.008	0.4 ± 0.3
SG2 + SNV	0.974 ± 0.038	0.976 ± 0.035	0.971 ± 0.064	0.983 ± 0.037	0.999 ± 0.003	2.6 ± 0.9

Mean ± standard deviation values of classification metrics were obtained from fivefold cross-validation. The SVM_C_ column denotes the distribution (mean ± standard deviation) of the optimal regularization parameter values selected across folds for each preprocessing algorithm.

### Comparison of feature selection algorithms

**Fig. 2:**
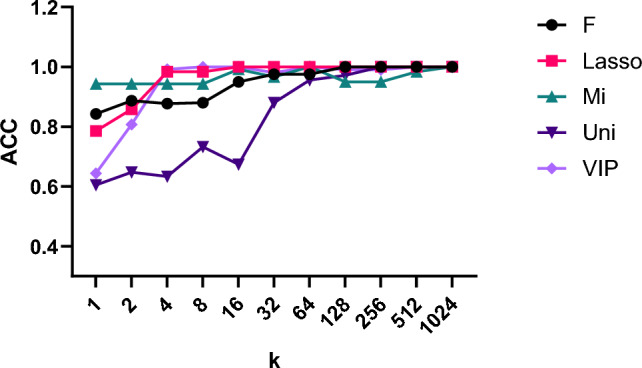
Discrimination accuracy of SVM classifiers under different feature-selection algorithms as a function of the number of selected spectral variables (bands; *k*). Accuracy (ACC) is shown for features selected by the F-statistic (F), least absolute shrinkage and selection operator (LASSO), mutual information (MI), univariate correlation (Uni), and variable importance in projection (VIP).

Figure 2 visualizes the SVM accuracy obtained when incrementally increasing the number of selected variables (k) for each feature selection algorithm. A comparison of feature selection algorithms under varying numbers of selected variables (k) revealed the superior performance of LASSO. As shown in Fig. 2, LASSO achieved higher accuracy than all other algorithms across almost all feature dimensions, reaching 0.918 ± 0.126 with only 2 bands and perfect classification at 64 bands (1.000 ± 0.000). By contrast, the F-statistic (F) and mutual information (Mi) approaches required at least 16 bands to achieve similar levels of performance; the univariate correlation (Uni) regression showed the weakest results across the entire range. VIP exhibited volatile behavior at low dimensions, with an accuracy of only 0.715 ± 0.112 using a single band and improved only gradually with additional bands. These findings indicate that LASSO not only provides the most robust and consistent feature selection but also enables accurate classification with a minimal number of spectral variables, underscoring its suitability as the primary feature selection strategy in this study.

### Integration of NIR and chemical data for practical applications

**Fig. 3:**
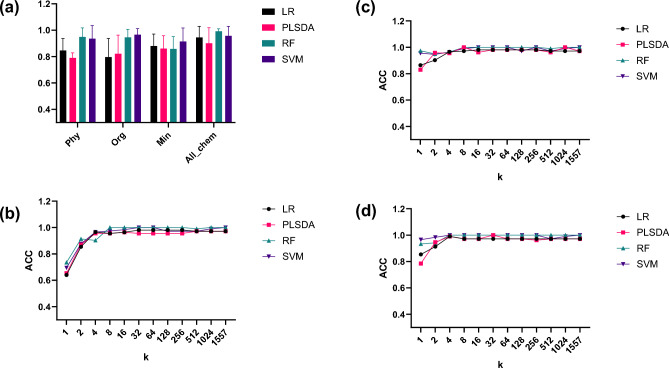
Model performance of chemical, near-infrared (NIR), and hybrid models across different feature dimensions. (**a**) Accuracy (ACC) of models using only physicochemical variables (color parameters, moisture, protein, fat, and ASTA value), organic acids (citric, malic, and fumaric acids), minerals, or combined chemistry-only variables. (**b**) Accuracy of models evaluated using NIR spectra using different numbers of selected bands (k). (**c**) Hybrid NIR + physicochemical (NIR + phy) models (spectral bands combined with physicochemical variables). (**d**) Hybrid NIR + organic acid (NIR + org) models (spectral bands combined with citric, malic, and fumaric acids).

When only chemical composition was used as the input variable, the binary origin discrimination accuracy was moderate across all models (Fig. 3a). All evaluated models (LR, PLS-DA, RF, SVM) are discriminant (hard-modelling) classifiers under a closed-set setting (domestic vs imported). The physicochemical data yielded accuracies ranging from 0.790 (PLS-DA) to 0.949 (RF), whereas the organic acid and mineral datasets showed similar or slightly higher performances. Combining all chemical variables improved the prediction, reaching 0.992 ± 0.019 with RF. These results indicate that although the chemical composition provides useful information for distinguishing sample origins, it is insufficient for consistently reliable origin classification.

In contrast, the NIR spectra alone produced markedly higher accuracy, particularly when more than four bands were included (Fig. 3b). At k = 4, both LR and SVM achieved 0.965 ± 0.055, and RF reached 0.902 ± 0.057. With 8–16 bands, RF and SVM attained 1.000 accuracy, and the performance remained stable even with further band expansion up to the full spectrum. This demonstrates that NIR alone is capable of near-perfect origin discrimination using a minimal number of spectral features. Nevertheless, because NIR bands are broad and overlapping and can be sensitive to scattering and measurement noise, hybrid models can provide an additional robustness margin by incorporating explicit compositional measurements.

When the NIR spectra were combined with physicochemical or organic acid data, the models consistently achieved robust results across all conditions. For NIR + phy (Fig. 3c), accuracies reached 1.000 with only 8 bands for RF and PLS-DA, while SVM maintained 0.990 ± 0.021. Similarly, NIR + org (Fig. 3d) reached perfect origin discrimination as early as k = 4 for both RF and SVM and remained stable across larger band dimensions. These findings indicate that while NIR alone provides sufficient potential to distinguish origins, the inclusion of complementary chemical data enhances the robustness and stability across different models and feature dimensions.

Collectively, these results outline practical deployment options. Chemical measurements alone provide informative but variable discrimination, while NIR offers rapid, low-cost, and nondestructive screening with strong classification performance. For industrial origin-label compliance screening—especially for chili powder used in kimchi—reliability and confidence are as important as accuracy. Therefore, integrating NIR with a small set of targeted chemical measurements provides added assurance and lowers misclassification risk, allowing stakeholders to select an approach aligned with their resources and required confidence level.

**Fig. 4:**
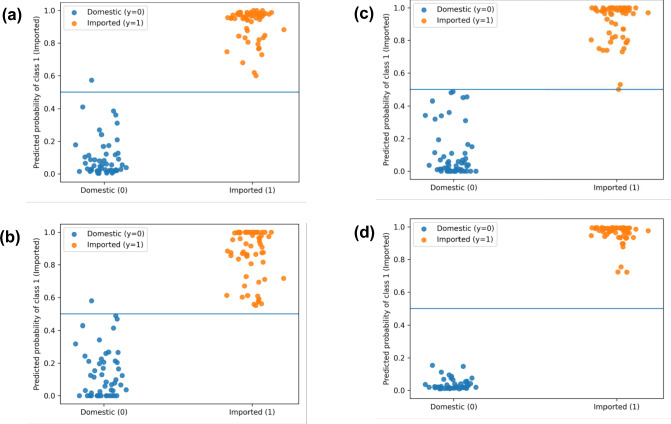
Out-of-fold discrimination plots for the best-performing hybrid setting (NIR + organic acid, LASSO k = 4), showing predicted probabilities of class 1 (Imported) for LR, PLS-DA, RF, and SVM (a–d) with a 0.5 decision threshold (horizontal line).

To visualize the discriminant decision behavior and explicitly indicate the decision thresholds, we aggregated and plotted out-of-fold predictions from the outer five-fold cross-validation for the best-performing setting (NIR + org with LASSO, k = 4 selected bands) across all models (Fig. 4). The predicted probability of the imported class is shown with a decision threshold of 0.5 (Fig. 4a–d). These plots demonstrate clear separation of the predefined classes under a closed-set binary discriminant (hard-modelling) setting.

### Feature stability and predicted-probability reliability of NIR-only and hybrid models

**Fig. 5:**
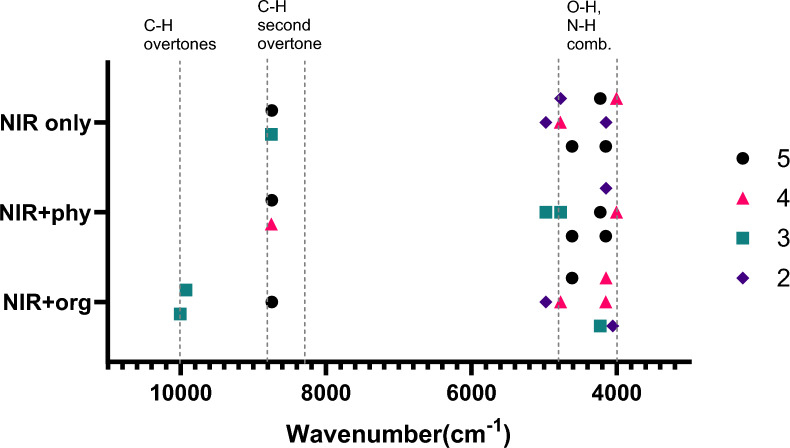
Stability of NIR band selection across cross-validation folds for the three optimal models (NIR-only, NIR + phy, and NIR + org). Each point represents a spectral variable selected in at least two folds, with the shape indicating the frequency of selection (2–5 folds).

Although the three optimal models (NIR-only, NIR + phy, and NIR + org) achieved nearly perfect accuracy, their internal behaviors revealed important differences. The band selection stability across cross-validation folds showed that certain NIR regions were consistently selected in the NIR-only model, suggesting the presence of robust spectral fingerprints for origin classification (Fig. 5). Notably, the most frequently selected bands were located near 4150–4800 cm^–1^, around 8740 cm^–1^, and occasionally close to the spectral edge at ∼10,000 cm^–1^. These regions were commonly assigned to the O–H combination bands of organic acids and water, the second overtone of the C–H stretching vibrations in lipids, and higher-order C–H overtones (Table S1).

The model predicted-probability reliability metrics further clarified these differences (Table 3). While all models achieved similarly low log-loss values, variations were evident in the ECE and Brier scores. The NIR + org model delivered perfect accuracy and the most reliable probability estimates (ECE = 0.006, Brier = 0.044), indicating highly reliable probability estimates. NIR-only also showed reasonably good probability reliability (ECE = 0.019, Brier = 0.073) despite a slightly lower accuracy. In contrast, although NIR + phy achieved perfect accuracy, it exhibited the least favorable probability reliability (ECE = 0.043, Brier = 0.172), suggesting that its probability estimates were less stable. The chemical-only model exhibited intermediate performance across all probability reliability metrics. These results demonstrated that the inclusion of organic acid variables enhanced the classification accuracy and provided the most trustworthy probability reliability, which is critical for industrial decision-making.

**Table 3:** Predicted-probability reliability metrics of different models (chemistry-only, NIR-only, NIR + phy, and NIR + org).

Metric	ACC	ECE@10	Brier	Log-loss
Chemistry-only	0.992 ± 0.019	0.040 ± 0.019	0.161 ± 0.053	0.483 ± 0.057
NIR-only	0.974 ± 0.038	0.019 ± 0.026	0.073 ± 0.066	0.488 ± 0.063
NIR + phy	1.000 ± 0.000	0.043 ± 0.032	0.172 ± 0.110	0.492 ± 0.065
NIR + org	1.000 ± 0.000	0.006 ± 0.009	0.044 ± 0.032	0.487 ± 0.058

Classification accuracy (ACC), expected calibration error (ECE@10), Brier score, and log-loss are reported as mean ± standard deviation across cross-validation folds. Lower ECE, Brier, and log-loss values indicate better probability reliability.

### Feature interpretability of NIR-only and hybrid models

**Fig. 6:**
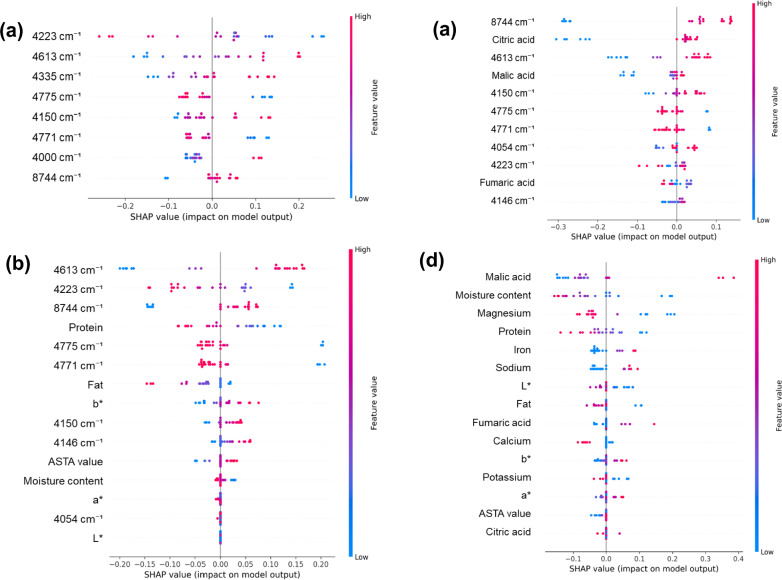
SHAP summary plots showing the most influential features for classification across models: (**a**) NIR-only, (**b**) NIR + org, (**c**) NIR + phy, and (**d**) chemistry-only.

SHAP analysis provided further interpretability by identifying the most influential features in each model (Fig. 6). In the NIR-only model (Fig. 6a), classification was primarily driven by spectral regions at 4223 cm^–1^, 4335 cm^–1^, 4613 cm^–1^, and 4771–4775 cm^–1^, corresponding to O–H combination bands and C–H overtones. A lipid-associated band at 8744 cm^–1^ also contributed, although its relative importance was lower than the other regions.

In the NIR + org model (Fig. 6b), citric acid and malic acid were the dominant predictors, reducing the importance of the O–H bands around 4200–4600 cm^–1^. This prominence of organic acids can be rationalized by post-harvest variability in dried pepper matrices, where malic/citric (and related) acids may shift with processing and storage. For example, during storage of pepper spices, malic acid has been reported to show an increasing trend, whereas citric acid tends to exhibit only minor or sample-dependent changes^28^. In addition, drying/thermal processing has been associated with decreases in citric acid alongside increases in malic and fumaric acids^29^, which is consistent with the contribution of fumaric acid observed here. Notably, the 8744 cm^–1^ band became more prominent in this hybrid model, which may indicate increased reliance on C–H-related spectral features (often linked to nonpolar constituents) once organic acids are provided explicitly. These patterns are consistent with the notion that geographic sourcing can be coupled with differences in cultivar and post-harvest handling—particularly drying and storage—which may co-vary with organic-acid profiles and color-related quality traits. Overall, these results highlight how the chemical analysis supports an interpretable compositional rationale for the hybrid model.

In the NIR + phy model (Fig. 6c), physicochemical variables, such as protein, fat, and moisture content, as well as color parameters (L*, a*, b*) and ASTA values, were ranked among the most influential features. This can be interpreted in light of prior reports showing that color attributes in dried pepper matrices are sensitive to variety and post-harvest processing conditions. For example, L* and a* values were significantly affected by sampling year, drying method, and variety, and drying technique was identified as a major driver of color variability in dried pepper matrices^29^. Moreover, prolonged sunlight exposure can substantially reduce ASTA color values, indicating that differences in drying/exposure conditions may translate into measurable shifts in ASTA-related quality traits^30^. Given that ASTA values are closely linked to pigment-related quality, the high SHAP ranking of ASTA and color parameters further supports the interpretation that the NIR + phy model leverages processing- and quality-associated signatures. Here, again, the 8744 cm^–1^ band showed higher importance than the NIR-only model, indicating that lipid-associated signals become more informative when integrated with compositional variables related to proximate analysis. The main NIR bands that were repeatedly selected across models, together with their tentative spectral assignments, are summarized in Table S1.

In contrast, the chemical-only model (Fig. 6d) relied exclusively on direct compositional measurements. Malic acid and moisture content were ranked among the most influential variables, followed by protein, minerals (e.g., magnesium (Mg), iron (Fe), sodium (Na), calcium (Ca), and potassium (K)), and colorimetric parameters (L*, a*, b*, and ASTA). Notably, the prominence of moisture content in the chemical-only model aligned with its significantly higher levels in the domestic samples (Table 1), which are indirectly represented by the O–H combination bands in the NIR-based models. Similarly, the high contribution of malic acid reflects its elevated concentration in imported samples, reinforcing its role as a key discriminator of geographic origin. The prominence of mineral variables (e.g., Mg, Fe, Na, Ca, and K) is in line with ICP-based multielement fingerprinting approaches reported for dry red pepper origin discrimination, where inorganic elements are interpreted as soil/environment-linked markers with comparatively stable profiles^10,31^. However, elemental profiling often requires dedicated ICP instrumentation and wet-digestion workflows, whereas the NIR + org strategy offers a more deployable screening framework while retaining chemical interpretability through organic-acid anchors.

Collectively, the SHAP results were consistent with the compositional contrasts in Table 1, providing a clear chemical rationale for model behavior. The three NIR-based approaches achieved comparably strong classification, but they differed in probability reliability and interpretability. The NIR-only model offers a simple, cost-effective screening option, whereas adding physicochemical or organic-acid variables improves interpretability; in particular, the NIR + org model provided a favorable balance between accuracy and probability reliability in our evaluation. Accordingly, while NIR-only solutions may be sufficient for rapid screening, integrating chemical variables—especially organic acids—can provide additional assurance in applications where transparent origin-label compliance screening is important, such as origin labeling of chili powder used in kimchi.

### Limitations and future works

This study has several limitations. First, the dataset consisted of 120 samples representing a binary comparison between domestic (Korean) and imported (Chinese and Vietnamese) chili powders. Although it included two production years and multiple cultivars, it does not fully reflect broader variability in growing regions, seasons, or processing conditions. Second, model performance was evaluated using internal cross-validation only, without an independent external validation set; therefore, the generalizability of the proposed framework remains to be verified. Third, the finding that four NIR bands yielded near-perfect classification accuracy should be interpreted within the context of the present dataset and acquisition conditions. Given the chemically complex and spectrally overlapping nature of chili powders, a fixed subset of wavelengths is unlikely to remain universally optimal across more diverse production environments.

Future work should therefore incorporate larger, multi-year datasets covering broader geographic and processing variation, and include independent external test sets to more rigorously assess model robustness. In addition, evaluating whether similarly compact spectral subsets remain effective under heterogeneous conditions will be important for establishing practical guidelines for industrial deployment.

## Conclusion

This study demonstrated that NIR spectroscopy, particularly when combined with chemical variables, provides a robust framework for classifying the origin of chili powder. Clear compositional contrasts were observed between the domestic and imported samples, including higher moisture, protein, and calcium in the domestic powders and elevated organic acids, sugars, and capsaicinoids in the imported powders. These differences were effectively captured by NIR spectra, where SG1 + SNV preprocessing and LASSO feature selection enabled near-perfect classification (NIR-only, ACC = 0.974 ± 0.038).

Hybrid models integrating chemical variables further improved performance, with both NIR + phy and NIR + org models achieving perfect accuracy (ACC = 1.000 ± 0.000). Notably, the NIR + org model provided the most reliable probability estimates (ECE = 0.006–0.009; Brier = 0.044–0.032), underscoring its reliability for practical applications. SHAP analysis confirmed that the predictive power was grounded in genuine compositional drivers, with organic acids, proteins, and moisture emerging alongside the O–H and C–H spectral bands as key discriminators. The explicit assessment of probability reliability and the use of SHAP-based interpretability distinguish this framework from many previous applications of NIR and chemometrics to chili powder origin, and are particularly important for regulatory and industrial decision-making.

Although this study focused on a binary domestic–imported classification, the proposed framework provides a basis for future extensions to finer regional distinctions using larger and more diverse datasets. Overall, the integration of NIR spectroscopy with targeted chemical profiling offers a rapid, nondestructive, and scalable solution for chili-powder origin-label compliance screening, supporting both consumer confidence and regulatory compliance.

## Materials and methods

### Sample collection and preparation

A total of 120 chili powder samples were analyzed, including 54 domestic products produced in Korea and 66 imported products from China and Vietnam. All samples were provided by the Rural Development Administration (Republic of Korea) in two separate batches collected in 2022 and 2023, reflecting the products distributed over different production years. Each sample was delivered in sealed packaging to prevent moisture exchange and stored at –20 °C immediately upon receipt.

For the analysis, the powders were brought to ambient conditions (approximately 25 °C, 40–50% RH) and equilibrated for at least 1 h prior to measurement. Approximately 5 g of powder was allocated for chemical assays, while separate portions were used for spectral measurements to avoid repeated freeze–thaw cycles.

### Chemical composition analysis

#### Composition and color

Moisture, protein, fat, and ash contents were determined following the official AOAC methods^32^ using standard gravimetric or Kjeldahl-based procedures. The colorimetric parameters (L*, a*, and b*) were measured using a calibrated colorimeter (CR-400, Konica Minolta Inc., Osaka, Japan)^33^.

#### Capsaicinoids

Capsaicin and dihydrocapsaicin were extracted and quantified following previously reported HPLC-based methods^34,35^ with modifications. Briefly, homogenized samples (2.5 g) were mixed with 15 mL of methanol and glass beads in sealed vials, incubated at 90 °C for 1 h, and filtered through 0.2-μm membranes. The extracts were analyzed using an Agilent 1260 Infinity HPLC system equipped with a fluorescence detector (Ex 280 nm, Em 325 nm) and a C18 column (Lachrom Ultra, 50 × 2.0 mm, 2 μm; Hitachi, Japan). The mobile phase consisted of 0.1% acetic acid (A) and acetonitrile (B) in an isocratic ratio of 60:40, with a flow rate of 0.6 mL/min and an injection volume of 2 μL.

#### Organic acids

Organic acid extraction and analysis were performed according to previously reported methods^34^, with minor modifications. Briefly, 2 g of each sample was extracted with 50 mL of distilled water for 30 min in a sonicator, filtered, and injected (10 μL) into an HPLC system (1260 Infinity, Agilent Technologies, USA) equipped with a diode array detector at 210 nm. Separation was achieved on an Aminex HPX-87H column (300 × 7.8 mm, 9 μm; Bio-Rad, USA) maintained at 50 °C, using 0.008 N H₂SO₄ as the mobile phase under isocratic conditions (flow 0.6 mL/min, 30 min). Citric, malic, and fumaric acids were identified by comparison with reference standards and were quantified using calibration curves.

#### Sugars

Free sugar analysis was conducted according to established protocols^36^ with minor modifications. Samples were heated in a water bath at 85 °C for 25 min, cooled, and filtered through a 0.45 μm nylon membrane. Glucose, fructose, sucrose, and total sugars were quantified using HPLC (1260 Infinity, Agilent Technologies, USA) equipped with a refractive index detector and a carbohydrate column (Asahipak NH₂P-50 4E, Shodex, Tokyo, Japan) maintained at 30 °C. The mobile phase consisted of 75% acetonitrile in water, delivered isocratically at 1.0 mL/min, with an injection volume of 6 μL. Calibration curves for individual sugars were prepared using reference standards.

#### Minerals

Mineral content was determined as previously described by Lee, et al.^37^. Approximately 1–2 g of each sample was digested in a polytetrafluoroethylene vessel using a microwave digestion system with 7 mL of nitric acid (70%, Dong Woo Fine Chem. Co., South Korea) and 1 mL of hydrogen peroxide (30%, Dong Woo Fine Chem. Co., South Korea). Calcium (Ca), iron (Fe), potassium (K), magnesium (Mg), manganese (Mn), phosphorus (P), zinc (Zn), and sodium (Na) were quantified using an ICP-OES system (Optima 8300, PerkinElmer, USA) and trace elements including lead (Pb) and cadmium (Cd) were analyzed using an ICP-MS system (NexION 300D, PerkinElmer, USA).

Calibration curves were prepared from certified multi-element standards (PerkinElmer, USA), and ultrapure water (18.2 MΩ·cm) was used throughout.

All measurements were performed in triplicate and expressed on a dry-weight basis.

### NIR spectroscopy

The NIR spectra of chili powder samples were collected using a Fourier-transform NIR spectrometer (Antaris II, Thermo Fisher Scientific Inc., Waltham, MA, USA) equipped with an integrating sphere for diffuse reflectance measurements, following the procedures of Kim and Ha^38^ and Yang, et al.^39^ with minor modifications. Spectral data were acquired over the range of 10,000–4000 cm^–1^ with a resolution of 4 cm^–1^, averaging 32 scans per sample to improve signal-to-noise ratio. Approximately 3 g of each sample was evenly loaded into a quartz sample cup and gently leveled to ensure a uniform surface without compaction. Three replicate spectra were recorded from independently prepared portions of each sample, and the mean spectrum was used for further analysis. The instrument performance was validated daily using a polystyrene standard, and background spectra were collected before each measurement series.

### Spectral preprocessing

To reduce the baseline shifts and scattering effects inherent in powdered samples, several spectral preprocessing algorithms were applied prior to modeling. Savitzky–Golay (SG) smoothing with first- and second-order derivatives (SG1 and SG2; window size = 15 points, polynomial order = 2) was employed to enhance the resolution of the overlapping absorption bands. Standard normal variate (SNV) transformation and multiplicative scatter correction (MSC) were also applied, either individually or in combination with SG smoothing (e.g., SG0 + SNV, SG2 + SNV, and MA7 + SNV, where MA7 denotes a 7-point moving average). The raw spectra were retained for baseline comparisons. All preprocessing procedures were implemented in Python (v3.9) using in-house scripts and the scikit-learn library. These approaches are consistent with widely adopted practices in NIR spectroscopy preprocessing^40,41^, ensuring comparability with previous studies.

### Feature selection

Variable selection is a critical step in spectroscopic modeling, and a range of statistical and machine learning–based approaches have been widely applied in previous studies^42^. To identify the most informative spectral variables and reduce model dimensionality, five feature-selection algorithms were compared: Least absolute shrinkage and selection operator (LASSO), F-statistics, mutual information, univariate correlation, and variable importance in projection (VIP) derived from partial least squares (PLS). For LASSO, the regularization parameter (λ) was optimized by inner cross-validation to ensure stable shrinkage behavior. For each feature selection algorithm, we ranked all spectral variables and then trained SVM models using the top-k bands, where k was varied from 1 to 1024. The resulting origin discrimination accuracies as a function of k are summarized in Fig. 2, allowing a direct comparison of the dimensionality–performance trade-offs across algorithms. The selected features were then used as inputs for the subsequent analysis.

In this study, four modeling strategies were constructed: NIR-only (spectral bands only), NIR + phy (spectral bands combined with physicochemical variables, including color parameters, moisture, protein, fat, and ASTA value), NIR + org (spectral bands combined with citric, malic, and fumaric acids), and chemistry-only models (using only compositional variables without NIR information).

### Discriminant (hard-modelling) classifiers for origin discrimination

Various supervised machine learning classifiers have been widely applied in NIR spectroscopy for food origin and quality discrimination^42^. Based on previous studies, we selected three supervised discriminant (hard-modelling) classifiers—SVM, random forest (RF), and logistic regression (LR)— together with a traditional chemometric method, partial least squares discriminant analysis (PLS-DA), to evaluate the discrimination performance for chili powder samples. This study addresses a closed-set, supervised binary origin-discrimination problem (domestic vs imported), assuming that each sample belongs to one of the two predefined classes.

- **SVM**: A radial basis function kernel was used. The regularization parameter (*C*) and kernel width (γ) were tuned by grid search (C = 0.3, 1, 3; γ = {scale, 0.01, 0.001}).
- **RF**: Models were trained with varying numbers of trees (n_estimators = 100, 200, and 500) and maximum depths (max_depth = 10, none), with the optimal configuration selected based on cross-validation accuracy.
- **LR**: Implemented with L2 regularization. The regularization strength (*C*) was tuned across {0.5, 1, 3} using the “lbfgs” solver.
- **PLS-DA**: The number of latent variables was optimized by cross-validation from 2 to 15, subject to the dataset dimensionality constraints.

The model performance was assessed using stratified five-fold cross-validation. The performance metrics included classification accuracy (ACC), F1 score, precision, recall, and area under the receiver operating characteristic curve (AUC). In addition to these conventional metrics, predicted-probability reliability was evaluated to assess how well predicted probabilities agree with empirical outcome frequencies (i.e., reliability of probabilistic outputs), rather than classification alone^43^. Predicted-probability reliability was quantified using the expected calibration error (ECE), Brier score, and log loss defined as follows:

1 $$\mathrm{ACC}=\frac{TP+TN}{TP+TN+FP+FN}$$

2 $$\mathrm{Precision}=\frac{TP}{TP+FP}$$

3 $$\mathrm{Recall}=\frac{TP}{TP+FN}$$

4 $$\mathrm{F}1=\frac{2\cdot Precision\cdot Recall}{Precision+Recall}$$

5 $$\mathrm{ECE}=\sum_{m=1}^{M}\frac{\left|{B}_{m}\right|}{n}\left|acc \left({B}_{m}\right)-conf \left({B}_{m}\right)\right|$$

6 $$\mathrm{Brier}=\frac{1}{n}\sum_{i=1}^{n}{\left({y}_{i}-\widehat{{p}_{i}}\right)}^{2}$$

7 $$\mathrm{LogLoss}=-\frac{1}{n}\sum_{i=1}^{n}\left[{y}_{i}\mathrm{log}\left(\widehat{{p}_{i}}\right)+\left(1-{y}_{i}\right)\mathrm{log}\left(1-\widehat{{p}_{i}}\right)\right],$$

where TP, TN, FP, and FN denote true positives, true negatives, false positives, and false negatives, respectively; *B*_*m*_​ is the set of samples in bin *m*; *acc*(*B*_*m*_) and *conf*(*B*_*m*_) represent the empirical accuracy and the mean predicted confidence in bin *m*, respectively; and *p*_*i*_ denotes the predicted probability for sample *i*.

### Model interpretability

To enhance the transparency of the classification models, SHAP analysis was employed to quantify the relative contributions of the individual features. SHAP values were calculated for both the spectral and chemical composition variables to determine their influence on the prediction outcomes. This approach, which has been increasingly applied in food spectroscopy and chemometrics for model interpretation^44^, allows for a clear attribution of predictive importance to individual bands or compositional traits. For NIR-only models, the analysis highlighted key spectral regions consistently contributing to origin classification, whereas in the hybrid models, the relative importance of chemical variables, such as organic acids or proximate components, was examined.

By comparing these SHAP-derived rankings with baseline compositional differences, we ensured that the predictive performance was grounded in meaningful chemical variations rather than in spurious correlations. The SHAP values were computed using KernelExplainer, with up to 200 Monte Carlo samples per fold and a background sample size of 64. All interpretability analyses were conducted in Python using the SHAP library (v0.41.0).

### Statistical analysis

Comparisons between the domestic and imported chili powder samples were conducted using unpaired *t-*tests with Welch’s correction to account for unequal variance. To control for multiple testing across the 22 variables, the false discovery rate was set to Q = 1% using Benjamini, Krieger, and Yekutieli’s two-stage step-up method. Statistical analyses were performed using GraphPad Prism, version 10 (GraphPad Software, San Diego, CA, USA). Data are presented as mean ± standard deviation, with significance levels indicated as *p* < 0.05 (*), *p* < 0.01 (**), *p* < 0.001 (***), and not significant (ns).

## Role of the funding source

The funding bodies had no involvement in the study design, data collection, analysis, interpretation, or manuscript preparation.

## Supplementary Information

Supplementary Information.

## Data Availability

The data supporting the findings of this study are available from the corresponding author upon reasonable request.
