# Supplementary material for: Hybrid near-infrared and chemical-based machine learning enhances the reliability of chili powder origin classification
Source: Sci Rep. 2026 Apr 6;16:16533. doi: 10.1038/s41598-026-47486-7 (PMC13216347; doi:10.1038/s41598-026-47486-7)
Supplement: Supplementary file 1 — Supplementary Information. [file 41598_2026_47486_MOESM1_ESM.docx]

**Supplementary Material for:**

**Hybrid near-infrared and chemical-based machine learning enhances the reliability of chili powder origin classification**

Ji-Hee Yang^a, b†^, Hae-Il Yang^a†^, Se-Jin Park^a, b^, Sung-Gi Min^a^, Woo Jin Jun^b*^, and Young-Bae Chung^a*^

† These authors contributed equally to this work and share first authorship.

^a^Kimchi Factory Research Group, World Institute of Kimchi, Gwangju 61755, Republic of Korea

^b^ Division of Food and Nutrition, and Research Institute for Human Ecology, Chonnam National University, Gwangju 61186, Republic of Korea

***Corresponding author**

Woo Jin Jun. Division of Food and Nutrition, and Research Institute for Human Ecology, Chonnam National University, Gwangju 61186, Republic of Korea; Tel: +82-62-530-1337; Fax: +82-62-530-1339; E-mail: [wjjun@chonnam.ac.kr](mailto:wjjun@chonnam.ac.kr)

Young-Bae Chung. Kimchi Factory Research Group, World Institute of Kimchi, Gwangju 61755, Republic of Korea; Tel: +82-62-610-1734; Fax: +82-62-610-1850; E-mail: [ybchung@wikim.re.kr](mailto:ybchung@wikim.re.kr)

**1. Supplementary Figure:**

**Figure S1.** PCA score plot for country-level similarity assessment using standardized compositional variables.

**Table S1.** Major NIR bands contributing to origin classification and their tentative assignments (adapted from [Workman and Weyer^27^](#_ENREF_27)).

| Models | Wavenumber (cm⁻¹) | Tentative assignment |
| --- | --- | --- |
| NIR-only  NIR+phy  NIR+org | 4223 | OH/CH combination bands of water and hydroxyl groups in carbohydrates and organic acids. |
|  | 4613 | Intense OH combination band of liquid water and hydrogen-bonded hydroxyl groups, with additional CH combination features. |
|  | 4771–4775 | Upper OH/NH combination region; OH combination bands with minor CH contributions from carbohydrates and other organics. |
|  | 4150 | Lower edge of the OH/NH combination region; hydrogen-bonded OH and NH in moisture, proteins, and organic acids. |
|  | 8744 | Second overtones of CH₂/CH₃ stretching vibrations from lipids and other hydrophobic CH-rich components (e.g. pigments). |
| NIR+phy  NIR+org | 4146 | OH/NH combination bands with overlapping CH modes in complex organic matrices. |
|  | 4054 | Weak OH/CH combination bands near the short-wavelength edge; mainly carbohydrates and other CH-rich constituents. |
| NIR-only | 4335 | Mixed OH/NH/CH combination bands associated with water, carbohydrates, and proteinaceous components in plant matrices. |

**Table S2.** Country-level summary of key chemical and physicochemical variables for chili powder samples from Korea, China, and Vietnam.

|  | Korean (n = 54) | Chinese (n = 57) | Vietnamese (n = 9) |
| --- | --- | --- | --- |
| L* | 35.79 ± 2.01 | 35.68 ± 4.09 | 49.37 ± 1.31 |
| a* | 27.73 ± 5.05 | 30.14 ± 6.12 | 30.45 ± 0.87 |
| b* | 20.85 ± 8.88 | 26.17 ± 11.40 | 49.56 ± 4.60 |
| Moisture content (%) | 8.71 ± 0.92 | 7.09 ± 2.56 | 4.30 ± 0.86 |
| ASTA color | 101.67 ± 24.46 | 124.77 ± 38.05 | 51.20 ± 1.27 |
| Protein (mg/kg) | 15.67 ± 1.54 | 13.63 ± 1.81 | 13.72 ± 0.68 |
| Fat (mg/kg) | 10.23 ± 1.22 | 10.33 ± 2.40 | 15.29 ± 1.97 |
| Citric acid (g/kg) | 18.74 ± 18.17 | 35.54 ± 4.66 | 25.12 ± 2.76 |
| Malic acid (g/kg) | 7.81 ± 7.52 | 16.84 ± 5.76 | 5.19 ± 0.26 |
| Fumaric acid (mg/kg) | 128.04 ± 142.06 | 105.74 ± 86.68 | 67.54 ± 4.00 |
| Sucrose (g/kg) | 4.27 ± 1.61 | 2.68 ± 1.33 | 2.30 ± 1.08 |
| Glucose (g/kg) | 54.86 ± 19.24 | 80.91 ± 22.79 | 39.08 ± 2.79 |
| Fructose (g/kg) | 105.34 ± 28.73 | 135.91 ± 23.05 | 67.40 ± 1.01 |
| Total sugar (g/kg) | 164.47 ± 47.76 | 219.50 ± 42.94 | 108.77 ± 4.82 |
| Sodium (mg/100 g) | 11.16 ± 5.91 | 1,233.63 ± 1,147.85 | 1,043.98 ± 76.68 |
| Calcium (mg/100 g) | 1,354.86 ± 1,284.95 | 42.54 ± 46.66 | 2.39 ± 0.12 |
| Iron (mg/100 g) | 4.54 ± 0.87 | 39.57 ± 31.60 | 42.77 ± 2.49 |
| K (mg/100 g) | 1,656.39 ± 1,592.64 | 1,384.03 ± 1,420.57 | 84.22 ± 6.05 |
| Mg (mg/100 g) | 196.25 ± 10.81 | 85.81 ± 84.66 | 16.43 ± 12.40 |
| Capsaicin (g/kg) | 314.19 ± 414.47 | 453.78 ± 385.23 | 2,951.26 ± 31.31 |
| Dihydrocapsaicin (g/kg) | 235.33 ± 252.25 | 298.38 ± 312.03 | 2,109.84 ± 298.74 |
| Total capsaicin (g/kg) | 549.53 ± 633.93 | 752.15 ± 666.46 | 5,061.10 ± 270.02 |

Values are expressed as mean ± standard deviation.
